# Structure of Weekly and Daily Physical Activity of Adults with Moderate and Severe Intellectual Disabilities in the Context of Barriers in Accessibility: A Preliminary Study

**DOI:** 10.3390/brainsci16010049

**Published:** 2025-12-29

**Authors:** Diana Celebańska, Barbara Rosołek, Bartłomiej Szreniawa, Anna Zwierzchowska

**Affiliations:** 1 Institute of Sport Sciences, The Jerzy Kukuczka Academy of Physical Education, 40-065 Katowice, Poland; b.rosolek@awf.katowice.pl (B.R.); a.zwierzchowska@awf.katowice.pl (A.Z.); 2 Department of Physical Activity and Health Promotion, The Jerzy Kukuczka Academy of Physical Education, 40-065 Katowice, Poland; b.szreniawa@awf.katowice.pl

**Keywords:** intellectual disability, occupational therapy workshops, physical activity, barriers

## Abstract

**Background**: The purpose of the study was to assess the level of physical activity (PA) and identify barriers to PA for adults with intellectual disabilities attending occupational therapy workshops (OTW). **Method**: Forty-three people participated in the study. PA level (step count) was evaluated using the Garmin Vivofit 4. Barriers were identified using the Barriers to Being Active Quiz. **Results**: The mean weekly steps were 10,581 ± 3981. Respondents were significantly more active on weekdays compared to weekends (*p* < 0.0002). During their stay at OTW, the participants took an average of 4257 steps, which was 40% of the mean step count on weekdays. Among the identified barriers to undertaking PA, lack of willpower and lack of skills were most frequently cited among the respondents. **Conclusions**: The level of PA was relatively high and showed great individual variation. The high level of activity during the participants’ stays in OTW indicates important role of these workshops in increasing PA in adults with intellectual disability.

## 1. Introduction

Physical activity (PA) is an important and modifiable component of lifestyle, and its benefits are well-documented [1] and include physical [2,3] mental [4,5,6], and psychosocial [7] health. These benefits also apply to people with intellectual disabilities [8], for whom PA is also one of the forms of physical [8,9,10] and social rehabilitation. PA is also an element that prevents early aging and the associated frailty syndrome, which is particularly important in the population of people with intellectual disabilities. Studies have shown that aging processes begin earlier in these people compared to non-disabled people, i.e., at 40–50 years of age [11,12], and manifest themselves in visual, hearing, and musculoskeletal dysfunctions, dementia, and chronic diseases [13,14]. Robertson et al. [15] (p. 485) indicate unequivocally that ‘the single most effective way of improving the health of people with intellectual disability would be to increase levels of moderate or vigorous physical activity’.

However, the results of available studies indicate that adults with intellectual disabilities are less active compared to those who are non-disabled. Melville, Hamilton, Hankey, Miller, Boyle [16], and Dairo, Collett, Dawes, and Oskrochi [17] showed that compared to 30–47% of the general population, only 9% of adults with intellectual disabilities met the guidelines for undertaking PA. Furthermore, as many as 60% of participants with intellectual disabilities were characterized by a sedentary lifestyle [18,19,20,21]. PA has also been reported to decline with age [22,23].

PA among people with intellectual disability is determined by a fundamental problem, namely lower IQ, which is directly related to reduced motivation and consistency in action, often accompanied by being overweight or obese and having morphofunctional problems. This should be defined as the so-called internal barriers to PA. However, there are also limitations, barriers that are related to external conditions (environmental and organizational), such as a lack of programs that include people with intellectual disabilities for general PA, the lack of staff and equipment adapted to the needs of people with intellectual disabilities, and the high costs of participating in various forms of PA [24]. Shields, Synnot, and Barr [25] also identified personal (e.g., lack of self-confidence) and social (e.g., negative social perceptions) factors as limiting the participation of people with intellectual disabilities in PA. The authors explicitly point out that to increase the participation of people with intellectual disabilities in PA, it is necessary to remove barriers and build an intrinsic willingness to participate [26,27], with the family environment [25] and the immediate social environment playing a major role.

In the context of limitations in independent daily functioning [28] and the cognitive and emotional consequences of the disability [29], people with intellectual disabilities benefit from institutional support. In Poland, the occupational therapy workshops (OTWs) are an example, in line with basic social and vocational rehabilitation. OTWs are institutions that identify solutions that enable participants to enter the labor market. The workshops operate in the field of vocational rehabilitation in collaboration with other institutions offering vocational rehabilitation (e.g., the Institute for Vocational Activity, social cooperatives, and sheltered workshops). OTWs also offer psychological and physiotherapy support, and perform activation functions, contributing to an increase in PA levels [5,30,31]. Studies [5,32,33] consistently show that adults with intellectual disabilities achieve higher PA levels on weekdays, pointing to the crucial role of structured activities, such as OTW attendance.

Given the significance of PA for health and rehabilitation and the specific functioning of this population, it is necessary to accurately identify the structure of PA performed weekly, on weekdays, on weekends, and daily, with particular consideration for the time spent in OTWs. This will enable the identification of periods of lower PA and indicate barriers, which will ultimately allow for the development of effective, systemic solutions aimed at physical activation and social integration.

Our study presented here is a preliminary research report on the project “The City of Katowice: a friendly place for physical activity for adults with intellectual disabilities participating in occupational therapy workshops,” which was funded by the Upper Silesia and Zagłębie Metropolitan Region as part of the “Metropolitan Fund for Support of Science” program in 2022–2024 (in accordance with grant agreement No. RW/56/2023). The major aim of the project was to promote a healthy lifestyle among people with intellectual disabilities. All OTWs in the city of Katowice for people with intellectual disabilities were invited to participate in the project. Only four OTWs out of the six located in the Silesian agglomeration joined the cooperation.

In this study, the aim of our research was to assess the level of PA performed weekly (Mon–Sun), on weekdays (Mon–Fri), on weekends (Sat–Sun), and daily, including stays in OTWs, in adults with intellectual disabilities, and to identify barriers to undertaking PA in this group.

## 2. Materials and Methods

### 2.1. Participants

The studies were observational, quantitative, and qualitative. The study was conducted in June–July 2023. Purposive sampling was used in the study. Adults with moderate or severe intellectual disabilities, attending OTWs, who consented to participate (in the case of incapacitation, the consent was signed on behalf of the participant with intellectual disability by their legal guardian), and who walked independently were included.

The exclusion criteria were a lack of consent, a lack of orthopedic aids, and those who did not have any motor dysfunctions. We had informational and educational meetings with participants and their parents/legal guardians. Participants were familiarized with the study protocol, consent was obtained (from participants or, in the case of minors or legal incapacitation, from parents/legal guardians), and participants were informed of the possibility of withdrawal from the study at any stage.

Forty-three people (23 men and 20 women) with moderate (MO) (*n* = 21) and severe (SV) (*n* = 22) intellectual disabilities were studied. The mean age of the participants was 34.1 ± 6.9. Descriptive characteristics of participants are shown in Table 1.

Among physical parameters, there was significant variation between F and M for BH (*p* < 0.000). Individuals with an SV degree of intellectual disability had significantly higher mean BMI (*p* < 0.008) compared to those with an MO degree of intellectual disability. More than 50% of the participants were characterized by above-normal BMI, who were more often women and those with an SV degree of intellectual disability (see Table 1).

The research protocol was approved by the Bioethics Committee at the Jerzy Kukuczka Academy of Physical Education in Katowice, Poland (9/2012 of 8 March 2012 with annex KB/47/2022), and it met the ethical standards of the Declaration of Helsinki, 2013.

### 2.2. Measurements

The direct observation method was used in the study. All measurements were taken by one person using the tools and following the procedures of the certified Laboratory of Densitometry and Diagnostics of Body Structure, Composition, and Posture at the Jerzy Kukuczka Academy of Physical Education in Katowice, Institute of Healthy Living (PN-EN ISO 9001:2015 [34]).

Body height (BH) was measured twice using a Charder HM 200P stadiometer in the standing position. The higher result was recorded with an accuracy of 1 mm.

Body mass (BM) was measured using a TANITA MC 780 MAS (TANITA Corporation, Tokyo, Japan) with an accuracy of 0.1 kg.

Waist circumference (WC) was measured using a tape measure at the midpoint between the lower edge of the last palpable rib and the apex of the iliac crest, at the end of the expiratory phase, with an accuracy of 0.5 cm.

Hip circumference (HC) was measured using a tape measure placed parallel to the ground, taking into account the largest gluteal muscle circumference with an accuracy of 0.5 cm [35].

BMI (calculated according to the formula: BMI = body mass[kg]/body height^2^[m]) [36]. PA levels were measured as step count using a Garmin Vivofit 4 wristband, worn by the participants for 7 consecutive days on the wrist of their non-dominant hand. The results were read out and recorded on an observation sheet every evening before bedtime (by the respondent or a parent/legal guardian), and at the entrance to, and exit from, the OTW (by the physiotherapist working for the OTW). Both parents/legal guardians and physiotherapists were trained in the use of the wristband and familiarized themselves with the observation tables. The percentage of participants meeting PA recommendations was verified using the classification described by Tudor-Locke & Bassett [37] and Tudor-Locke, Johnson, and Katzmarzyk [38], according to which ˂5000 steps means sedentary, 5000–7499 means low active, 7500–9999 means somewhat active, 10,000–12,499 means active, and ≥12,500 means highly active. The desired level of PA that positively affects health was considered to be ≥10,000 steps/day [37].

Identification of barriers to undertaking PA by people with intellectual disabilities was based on the Barriers to Being Active Quiz: What keeps you from being more active? questionnaire, which respondents completed with the assistance of a parent/legal guardian or a cooperating OTW physiotherapist. The questionnaire consists of 21 statements grouped into 7 categories of barriers to undertaking PA: (a) lack of time, (b) social influence, (c) lack of energy, (d) lack of willpower, (e) fear of injury, (f) lack of skill, and (g) lack of resources. Respondents rated the agreement with the statements (‘very likely’ = 3 points, ‘somewhat likely’ = 2 points, ‘somewhat unlikely’ = 1 point, and ‘very unlikely’ = 0 points), and the points were summed into categories. A score of 5 or above in any category shows that this is an important barrier.

### 2.3. Statistical Analysis

Data were collected and analyzed using Statistica v 13.3 software. For all quantitative variables, means ($\bar{x}$) and standard deviation (sd), and their variation by gender and degree of intellectual disability of the participants was verified (due to small group sizes, the Mann–Whitney U test was used). The variation in the level of PA (measured by step count) between weekdays (Mon–Fri) and weekends (Sat–Sun) was verified. The percentage of respondents daily PA completed during their stay in an OTW was calculated. The percentage of subjects meeting PA recommendations was calculated. The significance level was set at *p* < 0.05. Based on the records, the mean step count was calculated: weekly (Mon–Sun), on weekdays (Mon–Fri), on weekends (Sat–Sun), and daily, including step count in OTW.

## 3. Results

### 3.1. PA Level

The mean weekly step count of the participants with intellectual disabilities was 10,581. In total, respondents were more active on weekdays (Mon–Fri) than on weekends (Sat–Sun) (*p* < 0.0002), which was true for F (*p* < 0.02) and M (*p* < 0.005), and those with MO (*p* < 0.002) and SV (*p* < 0.04) degrees of intellectual disability. Furthermore, the average step count on weekdays for all participants, and by gender and degree of intellectual disability, exceeded 10,000, which was not observed on weekends. For daily activity, each working day, the participants reached a mean step count close to or above 10,000, and the day with the highest average step count was Tuesday.

M were characterized by a higher mean weekly step count, as well as that on weekdays and weekends. F were more active on Wednesdays and Fridays. The differences were not statistically significant.

Respondents with a moderate degree of intellectual disabilities were more active weekly and on weekdays. People with an SV degree of intellectual disabilities were more active on Fridays, Saturdays, and Sundays.

**Figure 1:**
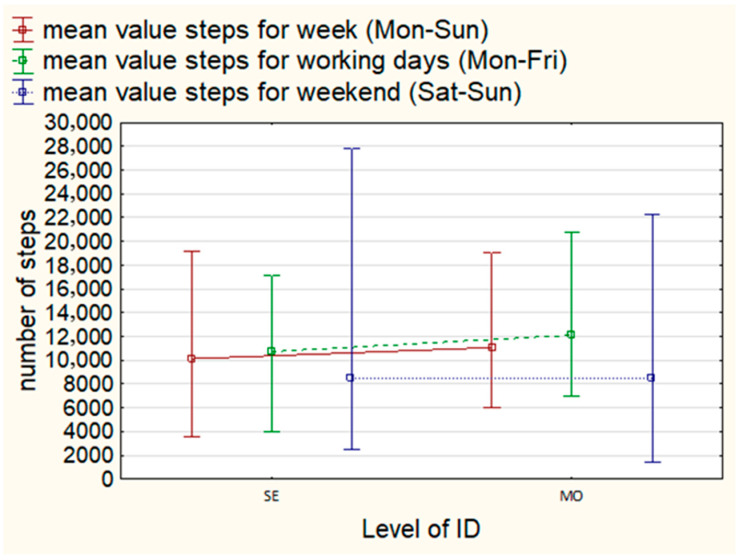
Mean, min, and max step count weekly, on weekdays, and on weekends by degree of disability.

**Figure 2:**
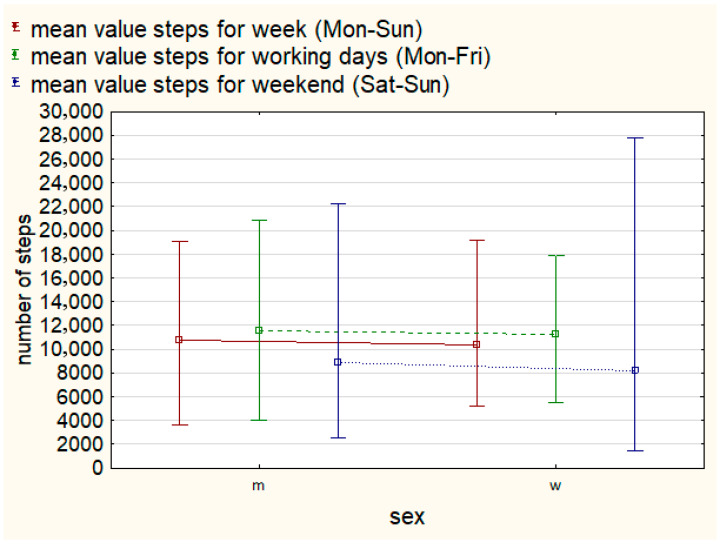
Mean, min, and max step count weekly, on weekdays, and on weekends by gender.

At the same time, the study group was characterized by a large internal variation in the level of PA measured by the step count, as evidenced by the relatively high values of standard deviations (see Table 2), as well as the minimum and maximum values of the average step count recorded weekly (Mon–Sun), on weekdays (Mon–Fri), and on weekends among F and M and people with MO and SV degrees of intellectual disabilities (Sat–Sun) (see Figure 1 and Figure 2).

**Figure 3:**
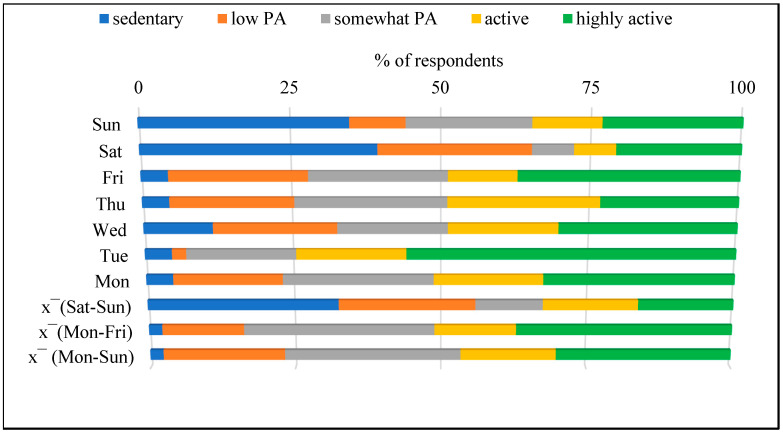
Qualitative assessment of the PA levels in participants. Note: $\overset{¯}{x}$—mean value, Mon—Monday, Tue—Tuesday, Wed—Wednesday, Thu—Thursday, Fri—Friday, Sat—Saturday, Sun—Sunday.

For the mean step count recorded weekly, 20 people (46.5%) were classified as active or highly active (>10,000 steps/day). The mean step count on weekdays allowed 22 people (51.1%) to be classified in this group, whereas on weekends, this was 14 people (32.6%). For step count recorded daily, only five of the respondents achieved active or highly active status 7 days a week (see Figure 3).

The respondents (*n* = 43) took an average of 4257.6 ± 2037.6 steps per day during their stay at OTWs (Mon–Fri), which was 40.2% of the mean step count on weekdays. Compared to women, men were more active daily during their stay in OTWs. Those with an MO degree of intellectual disability were more active than those with an SV degree of intellectual disability (Mon–Thu). The variation was not statistically significant (see Table 3).

### 3.2. Barriers to Undertaking PA

**Figure 4:**
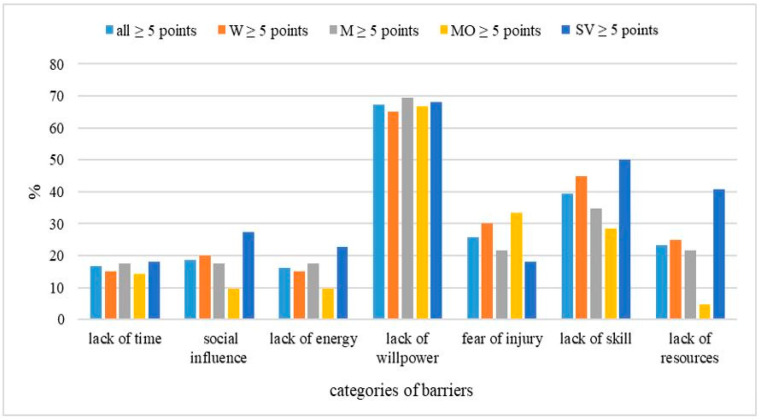
Percentage of total, W, M, and people with MO and SV degrees of intellectual disability who scored >5 points in each barrier category. Note: W—women, M—men, MO—moderate level of intellectual disability, SV—severe level of intellectual disability.

Based on the methodology used, a barrier category is considered significant if it scores ≥ 5 points. Among the most significant categories of barriers to undertaking PA by respondents were a lack of willpower (67.8%), lack of skills, fear of injuries, and lack of resources. This trend was also true for F and M. The participants with an MO degree of intellectual disability, among the four most significant barrier categories, indicated lack of willpower, lack of skills, fear of injuries, and lack of time, while those with an SV degree of intellectual disability indicated lack of willpower, lack of skills, and lack of resources (see Figure 4).

## 4. Discussion

The aim of the research was to assess the level of PA performed weekly (Mon–Sun), on weekdays (Mon–Fri), on weekends (Sat–Sun), and daily, including stays in OTWs in adults with intellectual disabilities, and to identify barriers to undertaking PA in this group. Individuals with intellectual disabilities are characterized by varying developmental potential, and their cognitive functions and processes deviate from the generally accepted normal levels [39], which are associated with difficulties in decision-making, managing their own lives [40], and exposure to isolation [41]. The results of the present study are consistent with this perspective, as evidenced by the considerable variability in PA levels observed across the week. Although the mean weekly step count exceeded 10,000 steps, large standard deviations and wide ranges of minimum and maximum values indicate substantial inter-individual differences in PA patterns. Moreover, a clear weekday–weekend discrepancy was observed, with significantly higher PA levels recorded on weekdays than on weekends, suggesting the importance of structured daily routines in supporting PA engagement among adults with intellectual disabilities.

General trends in studies of PA levels of adults with intellectual disabilities indicate lower levels compared to the general population [42,43,44]. Temple, Frey, and Stanish [45] found that about two-thirds of adults with disabilities have not achieved the minimum level of PA to provide health benefits. Stanish & Draheim [46] and Hilgenkamp, Reis, van Wijck, and Evenhuis [47] demonstrated that about 17–21% of adults and older adults with intellectual disabilities took the recommended 10,000 steps per day. In our study, the percentage was 46.5%, which is a relatively high result. However, it should be noted that the results obtained by individuals varied greatly, with the mean weekly step count ranging from 3578 to 19,135. People with intellectual disabilities are a highly heterogeneous group [39], and this heterogeneity relates to physical, cognitive, and social development, as well as the level of independence in daily functioning, which translates into differences in PA levels.

When we compared participants with moderate (MO) and severe (SV) degrees of intellectual disability, individuals with an MO degree tended to demonstrate higher mean weekly and weekday step counts, whereas those with an SV degree showed relatively higher activity levels during weekends. Although these differences did not reach statistical significance, the observed trend suggests that the degree of intellectual disability, and indirectly, cognitive functioning (IQ), may act as an internal factor limiting engagement in PA. The variability in PA levels observed in the present study appears to be closely linked to the barriers to PA reported by participants. Lack of willpower was the most frequently indicated barrier (67.8%), irrespective of gender and degree of intellectual disability, suggesting that motivational deficits constitute a central limitation to PA engagement in adults with intellectual disabilities. In this population, reduced motivation is likely associated with cognitive limitations, lower self-efficacy, and difficulties in initiating and sustaining goal-directed behaviors, particularly in unstructured contexts [39,41]. The co-occurrence of lack of skills and fear of injuries further indicates insufficient motor competence and limited confidence in performing PA safely, which may contribute to avoidance behaviors and reduced activity levels, especially outside supervised environments [48,49].

In addition to cognitive factors, internal limitations such as age, body mass index (BMI), and other somatic characteristics may further differentiate PA levels between individuals, although these variables were not the primary focus of the present analysis. Taken together, these factors can be described as internal barriers that may restrict participation in PA among adults with intellectual disabilities.

Differences in barrier profiles between individuals with moderate and severe intellectual disabilities provide additional context for the observed PA patterns. Participants with a moderate degree of intellectual disability more frequently reported lack of time as a significant barrier, which may reflect greater awareness of daily routines and competing obligations related to higher levels of independence and participation in organized activities, such as OTWs. In contrast, individuals with severe intellectual disability more often identified a lack of resources as a key barrier, likely reflecting greater dependence on external support and limited access to facilities or adapted equipment. These findings are consistent with the significantly higher PA levels recorded on weekdays compared to weekends, indicating that structured settings, such as OTWs, effectively reduce motivational and resource-related barriers. Conversely, during weekends, when external organization is limited, internal barriers appear to exert a stronger influence, leading to reduced PA engagement. This underscores the need for structured, supportive interventions that address both internal and external barriers and that are adapted to the cognitive and functional capacities of adults with intellectual disabilities [42,45].

Previous studies by Dixon-Ibarra, Lee, Dugala [48], and Vancampfort et al. [49] demonstrated that PA levels in people with more severe degrees of intellectual disability and older people are lower. A similar, yet not statistically significant, trend was observed in the present study, which is a limitation of the universality of conclusions. In our opinion, it was a consequence of too small a group. Furthermore, Gawlik et al. [10] and Oviedo et al. [21] reported a trend toward higher PA levels for men with intellectual disability, which was also reflected in our findings, with men showing higher mean step counts across weekly, weekday, and weekend analyses.

To the best of our knowledge, the originality of this study was the use of an innovative model in the analysis of data on PA among people with intellectual disabilities. The authors not only classified weekly PA (weekend/Monday–Friday), but also separated time spent in OTWs from daily activity.

The results showed that participants accumulated an average of approximately 4258 steps per day during their stay at OTWs, accounting for about 40% of their total weekday PA. In addition, the overall PA levels were significantly higher on weekdays, when participants attended OTWs, compared to weekends. This detailed analysis also allows us to identify the time of day and day of the week that offer the potential for increasing PA in people with intellectual disabilities. Therefore, planning activation interventions for this group may be more targeted based on these results. These results confirm the important role of OTWs in the physical activation of people with intellectual disabilities and also point to the objectives of activities in increasing the level of PA. Another form of support for people with intellectual disabilities is the Special Olympics (SO). Walsh et al. [42] found that people with intellectual disabilities engaged in the SO were characterized by higher levels of PA and better performance. The above examples of support for people with intellectual disabilities in raising PA involve systemic, organized solutions, taking into account a certain percentage of the population of people with intellectual disabilities. It should be borne in mind that not all people with intellectual disabilities benefit from this kind of support; they live in family homes on a daily basis, and thus, the level of their PA is unknown and may be even lower than observed in the research.

It should be noted, however, that such systemic and organized forms of support reach only a portion of the population of people with intellectual disabilities. Many people with intellectual disabilities live in family homes and do not participate in organized programs, and their level of PA may even be lower than that observed in research settings. This is why systemic support for this population should be multifaceted and address not only individuals with intellectual disabilities but also their parents or legal guardians, creating a behavior pattern. Such support should encompass emotional, informational, material, instrumental, and spiritual dimensions of family life [41]. By instrumental support, we mean “assistance through pointing out specific remedial actions or modeling them,” and, consequently, preparing people with intellectual disabilities for active life in society. In addition to parents/legal guardians and the immediate environment in which the person with intellectual disability functions, an important role is played by OTWs. They are one form of social, vocational, and health rehabilitation. Participants of OTWs with intellectual disabilities engage in tasks performed in workshops, including making household and ceramic goods, using computers, carpentry, and other activities, while preparing themselves for employment and independence. Furthermore, they participate in physical activities aimed at improving function and preparation for lifelong PA. Although due to the cognitive skills of people with intellectual disabilities and limited daily living skills, this goal is extremely difficult to achieve, motor activation of people with intellectual disabilities is extremely important.

## 5. Conclusions

This preliminary study examined the structure of PA in adults with moderate and severe intellectual disabilities across weekly, weekday, weekend, and daily timeframes, including activity accumulated during attendance at OTWs. The findings indicate significantly higher PA levels on weekdays compared to weekends, with approximately 40% of weekday step counts accrued during time spent in OTWs.

Despite relatively favorable mean activity levels, substantial inter-individual variability and a marked reduction in activity during weekends were observed. Key barriers to PA include a lack of willpower, a lack of skills, fear of injuries, and limited resources, with differences in barrier profiles depending on the degree of intellectual disability. These findings suggest that both internal (cognitive and motivational) and external (organizational and environmental) factors limit PA participation, particularly in unstructured contexts.

## 6. The Strengths and Limitations

The strengths of this study include the objective assessment of PA using step counts, the detailed temporal analysis of activity patterns, and the novel separation of activity performed during OTWs from total daily PA. Limitations include the small sample size, cross-sectional design, and the lack of a control group not attending OTWs, which limit generalizability and causal interpretation. Purposive sampling was used, which makes generalization difficult. Future studies employing longitudinal designs and larger samples are needed to confirm these findings and to develop targeted interventions that support sustained PA beyond structured rehabilitation settings.

## Figures and Tables

**Table 1:** Descriptive characteristics of participants.

Parameter	Total (*N* = 43) ± sd	W (*n* = 20) ± sd	M (*n* = 23) ± sd	MO (*n* = 21) ± sd	SV (*n* = 22) ± sd
age	34.12 ± 6.99	34.45 ± 7.19	33.83 ± 6.97	35.71 ± 7.42	41.0 ± 6.35
BH (cm)	163.96 ± 13.25	154.08 ± 9.29 *	172.55 ± 9.72 *	165.98 ± 11.8	162.03 ± 14.5
BM (kg)	71.33 ± 20.38	63.68 ± 15.53	77.99 ± 22.02	66.8 ± 22.1 *	75.67 ± 18.03 *
WC (cm)	90.48 ± 16.59	85.85 ± 13.82	94.5 ± 17.99	87.1 ± 17.9	93.7 ± 14.9
BMI (kg/m^2^)	26.51 ± 6.84	27.03 ± 7.38	26.06 ± 6.46	23.9 ± 6.02	29.01 ± 6.75
normal (%)	46.5	40.0	52.2	66.7	27.3
overweight (%)	30.2	40.0	21.7	19.0	40.9
obese (%)	23.3	20.0	26.1	14.3	31.8

BH—body height; BM—body mass; WC—waist circumference; BMI—body mass index; *N*, *n*—number of participants; M—mean; sd—standard deviation; * statistically significant differences, W—women, M—men, MO—moderate degree of intellectual disability, and SV—severe degree of intellectual disability.

**Table 2:** Physical activity (PA) levels on a weekly, weekday, weekend, and daily basis.

	Mon $\overset{¯}{x}\;\pm$ sd	Tue $\overset{¯}{x}\;\pm$ sd	Wed $\overset{¯}{x}\;\pm$ sd	Thu $\overset{¯}{x}\;\pm$ sd	Fri $\overset{¯}{x}\;\pm$ sd	Sat $\overset{¯}{x}\;\pm$ sd	Sun $\overset{¯}{x}\;\pm$ sd
Total (*N* = 43)	11,399.1 ± 5231.9	13,721.4 ± 4978.8	10,249.8 ± 4476.1	10,512.0 ± 4157.3	11,141.9 ± 5346.6	8410.5 ± 6448.6	8638.7 ± 6366.8
M (*n* = 23)	11,904.4 ± 5468.7	14,192.4 ± 5180.7	10,176.1 ± 4743.8	11,083.0 ± 4721.8	10,319.3 ± 4220.4	8893.7 ± 6067.9	8804.6 ± 6649.7
W (*n* = 20)	10,817.9 ± 5021.5	13,179.9 ± 4810.6	10,334.5 ± 4268.3	9855.3 ± 3397.0	12,088.1 ± 6387.9	7854.8 ± 6977.3	8447.9 ± 6191.5
MO (*n* = 21)	11,832.7 ± 5070.2	15,529.1 ± 3869.6	10,981.2 ± 5217.5	11,618.3 ± 4158.7	10,555.2 ± 4412.5	8403.4 ± 5844.6	8564.4 ± 6560.1
SV (*n* = 22)	10,985.1 ± 5467.7	11,996.0 ± 5378.7	9551.6 ± 3619.3	9455.9 ± 3962.6	11,702.1 ± 6160.9	8417.2 ± 7115.9	8709.7 ± 6330.5
Total	11,404.9 ± 4037.3 *					8524.6 ± 5787.9 *	
M	11,535.0 ± 4215.0 *					8849.1 ± 5470.6 *	
W	11,255.1 ± 3926.3 *					8151.4 ± 6255.0 *	
MO	12,103.3 ± 3853.7 *					8483.9 ± 5844.6 *	
SV	10,738.1 ± 4183.6 *					8563.5 ± 6399.2 *	
Total	10,581.9 ± 3981.2						
M	10,767.6 ± 4123.5						
W	10,368.4 ± 3906.3						
MO	11,069.2 ± 3669.5						
SV	10,116.8 ± 4290.9						

*N*, *n*—number of participants; M—male, and W—women; MO—moderate degree of intellectual disability; SV—severe degree of intellectual disability; $\overset{¯}{x}$—mean; sd—standard deviation; * statistically significant differences, Mon—Monday, Tue—Tuesday, Wed—Wednesday, Thu—Thursday, Fri—Friday, Sat—Saturday, and Sun—Sunday.

**Table 3:** Step count in OTW.

	$\mathrm{Mon}\;\overset{¯}{x}$/%	$\mathrm{Tue}\;\overset{¯}{x}$/%	$\mathrm{Wed}\;\overset{¯}{x}$/%	$\mathrm{Thu}\;\overset{¯}{x}$/%	$\mathrm{Fri}\;\overset{¯}{x}$/%
Total (*N* = 43)	3602.9/30.2	5771.8/41.8	3738.6/34.7	3943.7/37.1	4231.1/37.7
M	4329.7/34.5	5934.5/42.3	3754.1/34.4	4124.0/37.1	4297.8/40.1
W	2767.2/25.2	5584.6/41.2	3720.7/35.1	3736.4/37.0	4154.3/34.9
MO	4149.9/34.5	7173.6/46.3	4315.6/37.1	4649.8/38.8	3837.2/36.4
SV	3080.9/26.1	4433.6/37.4	3187.7/32.4	3269.7/35.4	4607.0/38.9

Mon—Monday, Tue—Tuesday, Wed—Wednesday, Thu—Thursday, Fri—Friday, M—male, and W—women; MO—moderate degree of intellectual disability; SV—severe degree of intellectual disability; $\overset{¯}{x}$—mean, and %—percentage of the number of daily steps.

## Data Availability

The original contributions presented in this study are included in the article. Further inquiries can be directed to the corresponding author.

## Abbreviations

The following abbreviations are used in this manuscript:

PA	Physical activity
OTW	Occupational therapy workshop
